# *Thaicypridopsis baimaii* sp. nov. (Crustacea: Ostracoda) from Western Thailand, with Discussion on Distribution and Ecology of the Genus

**DOI:** 10.3390/life16101728

**Published:** 2026-10-15

**Authors:** Sukonthip Savatenalinton

**Affiliations:** Department of Biology, Faculty of Science, Mahasarakham University, Maha Sarakham 44150, Thailand; sukonthip.s@msu.ac.th

**Keywords:** biodiversity, crustacean, geographic barrier, Southeast Asia, systematics

## Abstract

*Thaicypridopsis* is rare and endemic to Thailand, appearing as a monospecific genus since its discovery. The recent investigation of ostracods in northeast Thailand, together with the reexamination of collections from other parts, including the poorly surveyed western region, uncovers the second and new species of this genus, *T. baimaii* sp. nov. The unveiling not only allows reconsideration of generic and specific characters within the genus but also fills a gap in the ostracod diversity of Thailand. *Thaicypridopsis baimaii* sp. nov. described from freshwater in the west, primarily differs from *T. longispinosa* Savatenalinton, 2018 by the short and smooth spines on the valve surface, the protruded posterior extremity of the right valve, the β seta on the mandibular palp with a remarkably long flagellum-like tip, and the number of large modified bristles on the first and the second endites of the maxillula. The detailed examination of the valve surface in the present work sheds light on the affinity among the Cypridopsinae genera. This study also updates the localities and distribution map of *T. longispinosa* and reveals the different geographic dispersal of these two species. The frequency of occurrence, with a large number of sampled localities in the northeast in particular, allows further discussion on allopatric evolution and ecology of the genus. A key to the genera of the tribe Zonocypridini is provided.

## 1. Introduction

Ostracoda is a group of microcrustaceans that inhabit several types of habitats, including phytotelmata and semiterrestrial sites [1,2]. In non-marine environments, the global number of ostracod species is 2420; most of them belong to Cyprididae [3]. In this family, Cypridopsinae Kaufmann, 1900 occupies large proportion in which many new taxa at different taxonomic categories (species, genera or even tribes) have been introduced in last five years, such as *Potamocypris meissneri* Szwarc et al., 2021, *Sarscypridopsis harundineti* Szwarc et al., 2021, *Cypridopsis schwartzi* Külköylüoğlu et al., 2022, *Sarscypridopsis denticulata* Smith and Ozawa, 2023, *Brasilodopsis* Almeida et al., 2021, *Paranadopsis* Almeida et al., 2021, *Songkhramodopsis* Savatenalinton, 2023, and Plesiocypridopsini Jacobs & Martens, 2022 [4,5,6,7,8,9]. Currently, Cypridopsinae contains 221 species and 29 genera [3,10]. They have been classified into seven tribes: Cyprettadopsini Savatenalinton, 2020, Cypridopsini Kaufmann, 1900, Paranadopsini Almeida et al., 2021, Plesiocypridini, Potamocypridini Ghetti & McKenzie, 1981, Songkhramodopsini Savatenalinton, 2023 and Zonocypridini Higuti & Martens, 2012 [3]. A key to tribes was provided by Savatenalinton [8]. In Thailand, ostracods have been explored in several kinds of habitats, both natural and man-made, ranging from (oxbow) lake, reservoir, swamp, pond, weirs, river, stream, (irrigation) canal and rice field. Most surveys were performed in the northern and northeastern parts, while other parts of the country (e.g., the west, central and south) have remained poorly studied. These studies discovered many new species and genera in several subfamilies, including Cypridopsinae, such as *Cyprettadopsis* Savatenalinton, 2020, *Mixtadopsis* Savatenalinton, 2025, *Neopotamocypris* Savatenalinton, 2024, *Siamopsis* Savatenalinton, 2017, *Songkhramodopsis* and *Thaicypridopsis* Savatenalinton, 2018 [10,11].

*Thaicypridopsis* is one of three genera of the tribe Zonocypridini, which is principally characterized by a large and strong serrated claw G2 on the female antenna [12]. In addition, *Thaicypridopsis* is diagnosed by the distinctive tubercles on the RV postero-ventral part, the last bristles on all three endites with hirsute endings and spatula-shaped apexes, and the h2 claw on the T2 with a bifurcate tip, as described by Savatenalinton [10]. Since then, it has been a monospecific genus. *Thaicypridopsis* exhibits several features differing from other cypridopsine genera and also shared characters with some members of Oncocypridinae, a questionable subfamily. Although Oncocypridinae presently belongs to Notodromadidae [3,13,14], some researchers proposed it as a tribe of Cypridopsinae [10,15].

Presently, the Thai ostracod fauna possesses 96 species [11,16]. However, the number is most likely underestimated, as it has been continuously increasing in the last 15 years. This also emphasizes that the actual species diversity of ostracods in Thailand is still unknown. Such a goal can be accomplished by surveys, step by step, especially in poorly studied areas/habitats in the northeast and other parts of the country. The recent investigation of ostracods in the northeast, together with the reexamination of ostracod collections from three other parts of Thailand (the north, the central and the west), uncovered a new species of the genus *Thaicypridopsis*, and thus the aim of the present paper is to describe this new species. In addition, a large number of sampled sites also allows a brief discussion on the distribution range and ecology of the *Thaicypridopsis* species.

## 2. Materials and Methods

Materials examined for the present work were obtained from the recent assemblage of the year 2026 and also from ostracod collections since 2005, which were supported by the Royal Belgian Institute of Natural Sciences, National Research Council of Thailand, Office of the Higher Education Commission, Thailand Research Fund, and Mahasarakham University. Samples were taken with a hand net (mesh size 200 μm), preserved with 70% ethanol in the field and, subsequently, ostracods were picked in the laboratory. Ostracods in Thailand were collected from several types of habitats, both natural and man-made, ranging from (oxbow) lakes, reservoirs, swamps, ponds, weirs, rivers, streams, (irrigation) canals, ditches and rice fields, in four parts of the country. The total number of surveyed localities was 424, arranged in central, northern, northeastern and western parts with the numbers 3, 54, 322 and 45, respectively. The water parameters (pH, Temp, and Cond) were measured by a multi-parameter water quality meter (Hanna HI98129—Hanna instruments, Inc., Port Louis, Mauritius).

Morphological study was performed via the following processes (see also [17]) prior to identification. Firstly, soft body parts and valves were split under a stereo-microscope (Olympus SZ40—Olympus Co., Ltd., Tokyo, Japan). Valves and carapaces were stored dry on micropalaeontological slides and then illustrated using a Scanning Electron Microscope (JEOL JSM6460LV—JEOL Ltd., Tokyo, Japan). Soft parts were dissected in glycerin on a glass slide, which were later sealed with nail polish. The illustrations of soft parts were done with the aid of a camera lucida attached to a compound microscope (Olympus BX-50—Olympus Co., Ltd., Tokyo, Japan). All types of materials are stored in the ostracod collection of the Faculty of Science, Mahasarakham University, Maha Sarakham, Thailand. The present work follows the model of limb chaetotaxy proposed by Broodbakker & Danielopol [18], together with the revised models for the second antenna by Martens [19,20] and for the third thoracopod by Meisch [21]. This research was approved by the Ethics Committee on Animal Care and Use of Mahasarakham University, Thailand (approval number IACUC-MSU-037-049/2026).

## 3. Results

### 3.1. Taxonomic Treatment

Superfamily Cypridoidea Baird, 1845.

Family Cyprididae Baird, 1845.

Subfamily Cypridopsinae Kaufmann, 1900.

#### 3.1.1. Tribe Zonocypridini Higuti & Martens, 2012

- Abbreviated diagnosis. LV overlapping RV, marginal septa absent, terminal segment of Mx1 palp cylindrical shape, A1 seven-segmented, female claw G2 robust and strongly serrated, T2 penultimate segment divided, T3 with pincer organ, CR reduced, flagellum-like.
- Genera included. *Cabelodopsis* Higuti & Martens, 2012, *Thaicypridopsis* Savatenalinton, 2018, *Zonocypris* G.W. Müller, 1898. A diagnostic key to genera is presented below.

Key to genera of the tribe Zonocypridini.

1 a. Large bristles on Mx1 endites with spatula-shaped apex, h2 claw on T2 with bifurcate tip, RV with distinctive submarginal tubercles on postero-ventral part…….………………………………………………………………………………..*Thaicypridopsis*

b. Large bristles on Mx1 endites without spatula-shaped apex, h2 claw on T2 without bifurcate tip, RV without distinctive submarginal tubercles on postero-ventral part……………………………………………………………………………………....….….….…. 2

2 a. Valve sculpture with prominent circular ridges, hinge well developed…….....…………………………………………………………………….……..……*Zonocypris*

b. Valve sculpture without prominent circular ridges, hinge simple and adont ..……………………………………………………………………….…….……….*Cabelodopsis*

#### 3.1.2. Genus Thaicypridopsis Savatenalinton, 2018

- Diagnosis (modified from Savatenalinton [11]). LV overlapping RV. RV in internal view with a row of submarginal tubercles, more distinctive on the postero-ventral part. Valve surface with spines, shallowly pitted, two types of setae (one type with a thick base and lateral tubercles on the pore rim). A2 with one strong serrated claw; G2 with concave tip. Largest seta on terminal segment of Md-palp apically equipped with hairs. Terminal segment of Mx1-palp cylindrical and elongated, largest setae (bristles) on all three endites with hirsute endings and spatula-shaped apexes. Seta d2 on T2 present (d1 absent), h2 claw with bifurcate ending. Terminal segment of T3 with apical pincer. Caudal ramus reduced, flagellum-like with triangular base.
- Species included. *Thaicypridopsis longispinosa* Savatenalinton, 2018 and *Thaicypridopsis baimaii* sp. nov.

### 3.2. New Taxon

**Figure 1:**
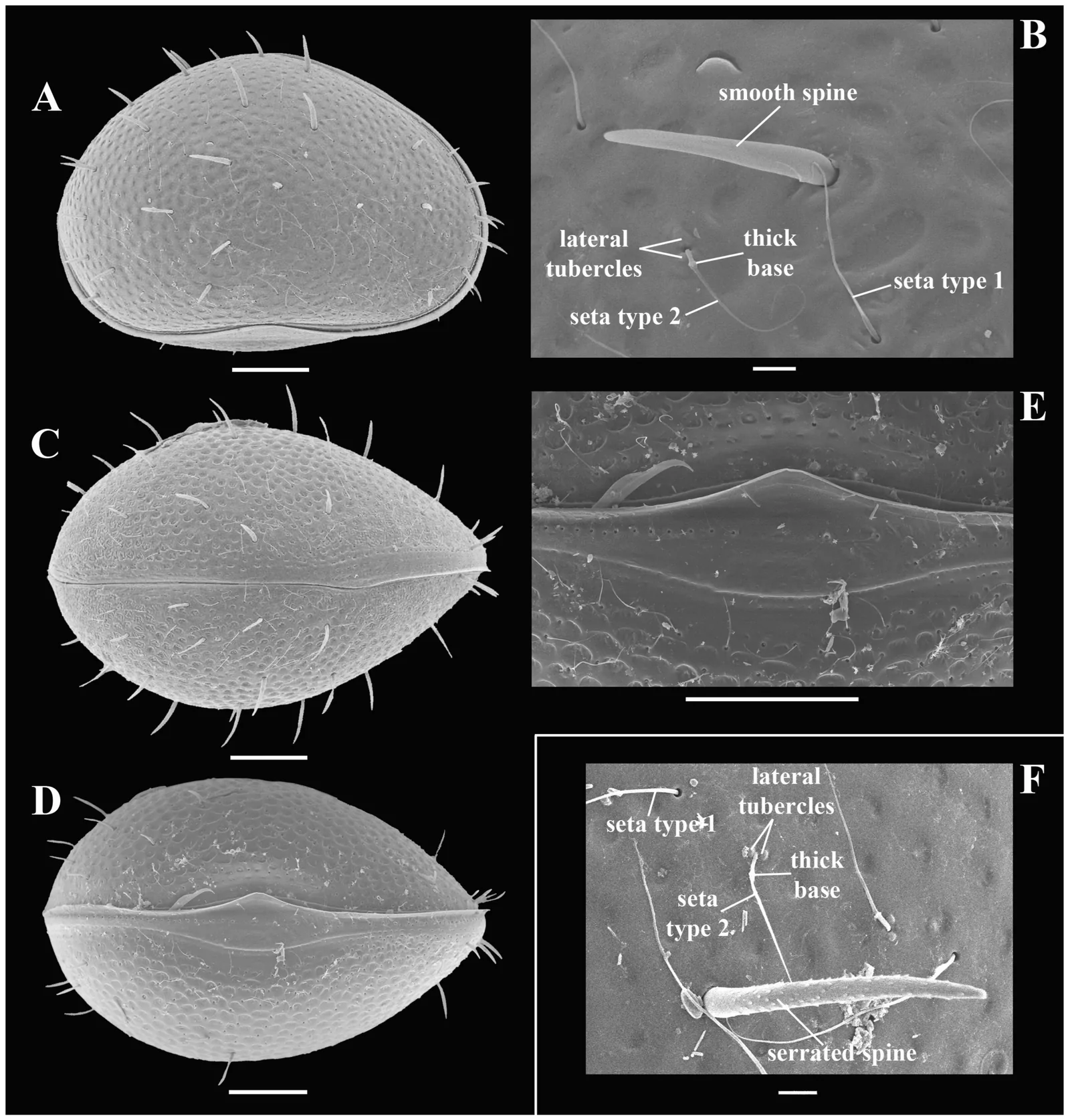
*Thaicypridopsis baimaii* sp. nov. (**A**–**E**). *Thaicypridopsis longispinosa* Savatenalinton, 2018 (**F**). (**A**) Cp in lateral view (MSU-ZOC.506). (**B**) Valve surface of RV (ditto). (**C**) Cp in dorsal view (MSU-ZOC.505). (**D**) Cp in ventral view (MSU-ZOC.501). (**E**) Middle part of Cp, ventral view (ditto). (**F**) Valve surface of RV (MSU-ZOC.257). Scale bars: 100 μm (**A**,**C**–**E**), 10 μm (**B**,**F**).

**Figure 2:**
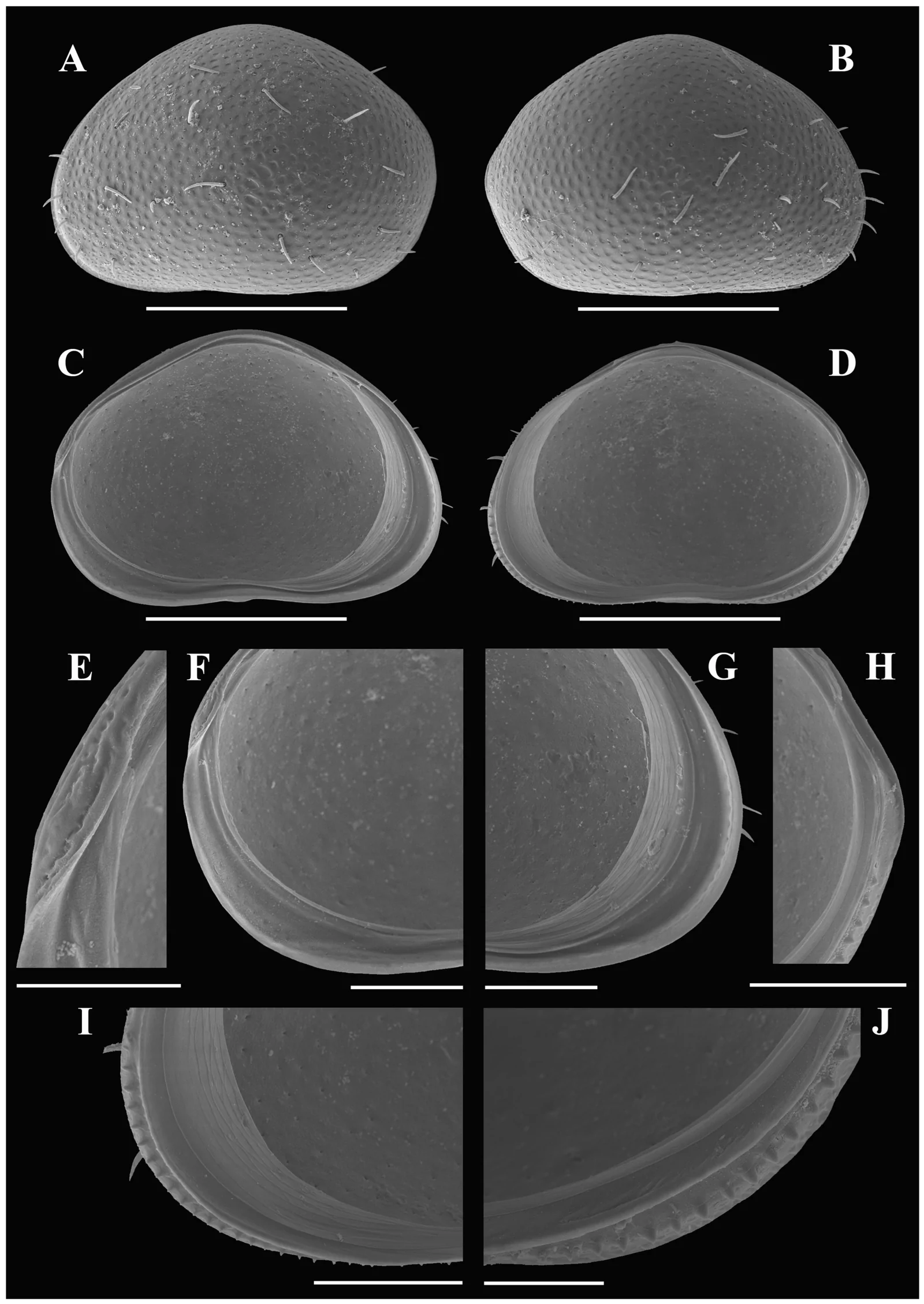
*Thaicypridopsis baimaii* sp. nov. (**A**) LV, external view (MSU-ZOC.502). (**B**) RV, external view (ditto). (**C**) LV, internal view (MSU-ZOC.500). (**D**) RV, internal view (ditto). (**E**) Postero-dorsal corner of LV, internal view (ditto). (**F**) Posterior part of LV, internal view (ditto). (**G**) Anterior part of LV, internal view (ditto). (**H**) Posterior tip of RV, internal view (ditto). (**I**) Antero-ventral part of RV, internal view (ditto). (**J**) Postero-ventral part of RV, internal view (ditto). Scale bars: 300 μm (**A**–**D**), 100 μm (**F**,**G**,**I**,**J**), 50 μm (**E**,**H**).

**Figure 3:**
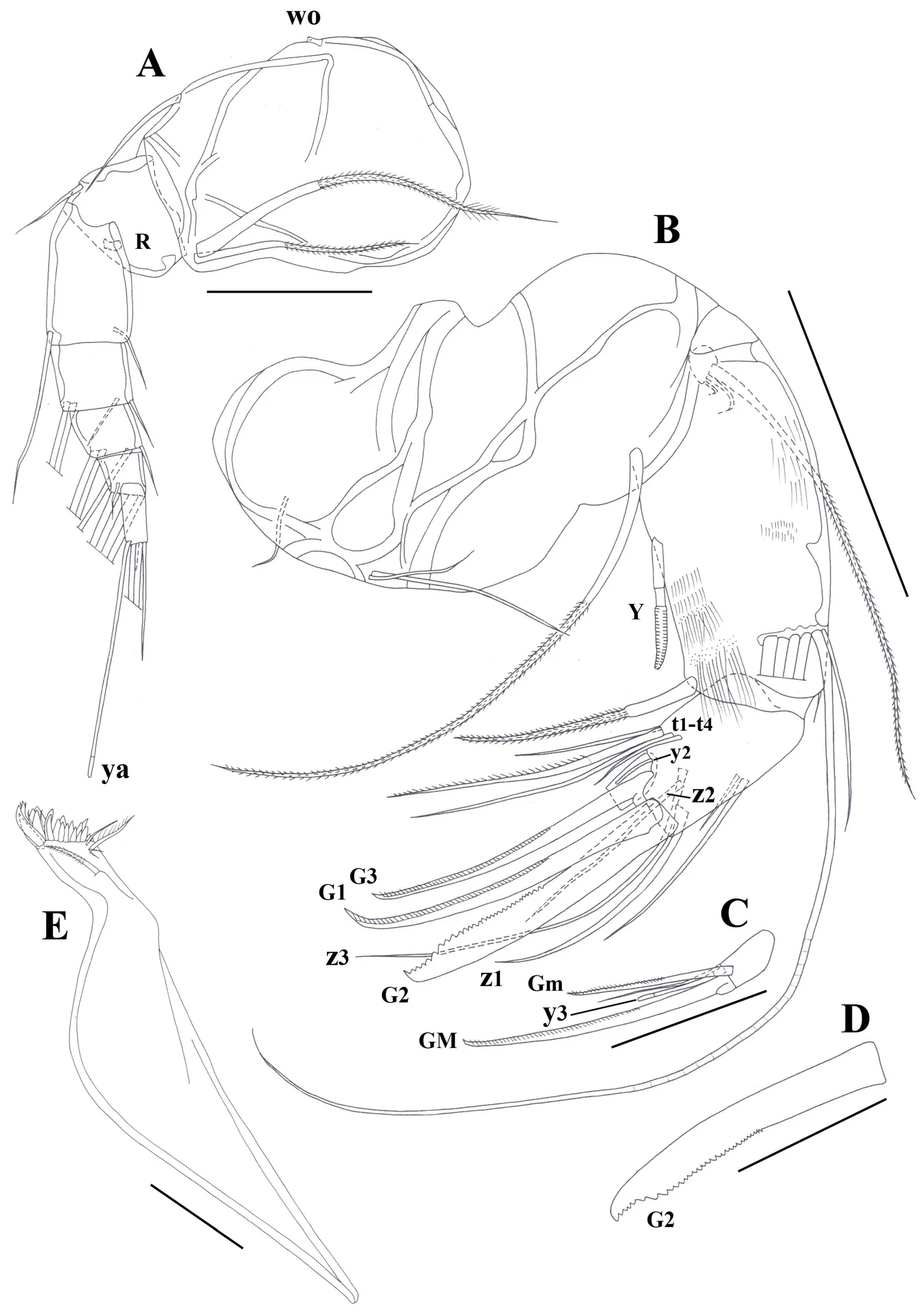
*Thaicypridopsis baimaii* sp. nov. (**A**) A1 (MSU-ZOC.512). (**B**) A2 (ditto). (**C**) Terminal segment of A2 (ditto). (**D**) Claw G2 of A2 (MSU-ZOC.508). (**E**) Md coxa (MSU-ZOC.512). Scale bars: 50 μm (**A**,**C**–**E**), 100 μm (**B**).

**Figure 4:**
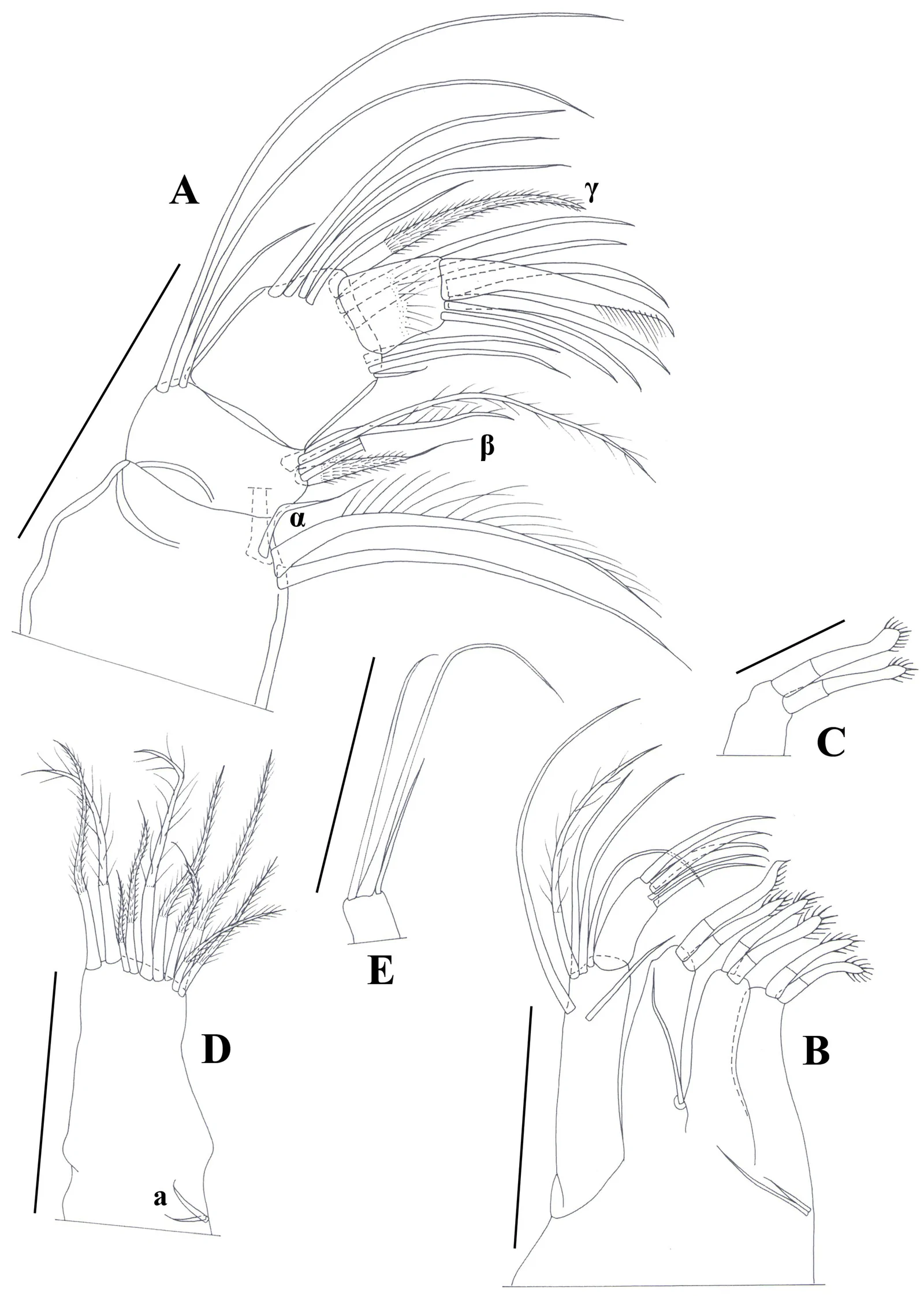
*Thaicypridopsis baimaii* sp. nov. (**A**) Md palp (MSU-ZOC.512). (**B**) Mx1 (ditto). (**C**) Two large bristles on third endite (MSU-ZOC.508). (**D**) T1 protopodite (ditto). (**E**) Tip of T1 palp (MSU-ZOC.512). Scale bars: 50 μm (**A**,**B**,**D**), 25 μm (**C**), 100 μm (**E**).

**Figure 5:**
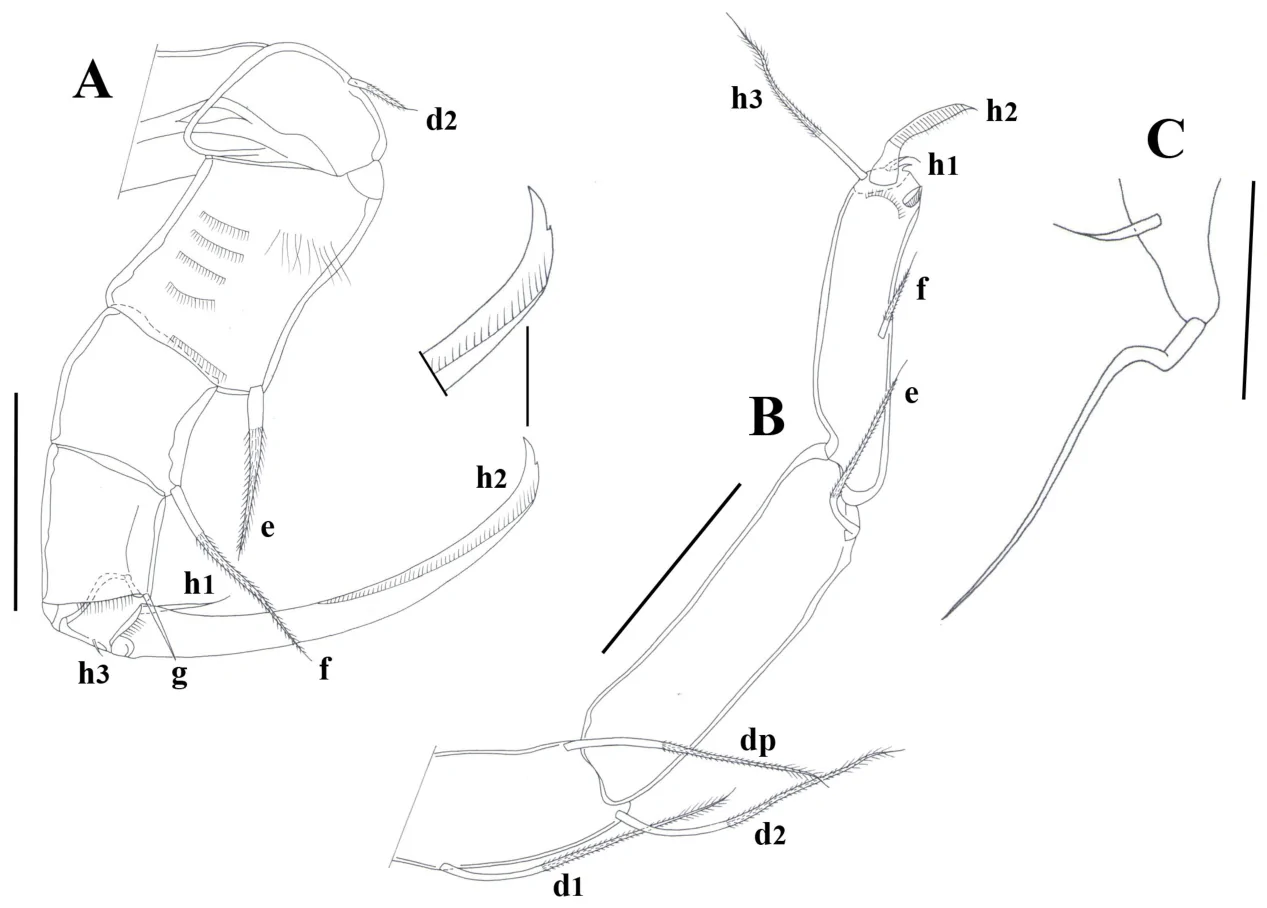
*Thaicypridopsis baimaii* sp. nov. (**A**) T2 (MSU-ZOC.512). (**B**) T3 (MSU-ZOC.500). (**C**) CR (ditto). Scale bars: 50 μm (**A**,**B**), 25 μm (**C**).

*Thaicypridopsis baimaii* sp. nov. (Figure 1, Figure 2, Figure 3, Figure 4 and Figure 5).

#### 3.2.1. Etymology

The species is named in honor of Prof. Dr. Visut Baimai (Mahidol University, Thailand), an outstanding biologist, in recognition of his prominent work on the evolution and systematics of mosquitoes and fruit flies in particular, and his long support for biodiversity studies in Thailand through the Biodiversity Research and Training Program (BRT).

#### 3.2.2. Diagnosis

Carapace in lateral view subtriangular with greatest height situated ca. mid-length, LV overlapping RV, all free margins (Figure 1A). Carapace in dorsal view subovate with maximum width situated slightly behind mid-length (Figure 1C). Valve surface with smooth spines dispersed and grounded with pits and two types of smooth setae (one without, another one with thick base), pore of the latter with two small lateral tubercles (Figure 1B). LV posterior part with double inner list, appearing well developed, but not oblique (Figure 2C,E). RV in internal view bearing tubercles along outer margin of inwardly displaced selvage anteriorly, ventrally, posteriorly, with more distinctive tubercles on antero-ventral and postero-ventral parts (Figure 2D,H,I). A1 seven-segmented (Figure 3A). Claw G2 on A2 strongly serrated with concave apex, natatory setae very long (far beyond end of terminal claw), four t-setae present in females with t2 claw-like appearance (Figure 3B,D). Terminal segment of Md palp with robust claw setting with hairy spicules, α seta smooth with flagellum-like tip, β seta plumose, cone-shaped with remarkably long, flagellum-like ending (Figure 4A). Terminal segment of Mx1 palp elongated, large bristle on endites claw-like with hirsute ending and spatula-shaped apex, number of large bristles on first to third endite being 2, 2, 2, respectively (Figure 4B,C). T1 with two a-setae (b-, c- and d-setae absent) (Figure 4D). Seta d2 on T2 short and thin (d1 absent); h2 claw with slightly bifurcate ending (Figure 5A). Caudal ramus reduced, flagellum-like with triangular base (Figure 5C).

#### 3.2.3. Type Materials and Repository

- Holotype. Female, soft parts dissected and preserved in glycerin on a slide, and valves stored dry in a micropalaeontological slide (MSU-ZOC.500).
- Paratypes. A total of 18 females, stored like the holotype (MSU-ZOC.502, 507 to 512, 517 to 518), Cp stored dry in a micropalaeontological slide (MSU-ZOC.501, 503 to 506, 513 to 516).
- Repository. The holotype and paratypes, including several individuals kept in 70% ethanol, are deposited in the ostracod collection of the Faculty of Science, Mahasarakham University, Maha Sarakham, Thailand.

#### 3.2.4. Localities

- Type locality. Nong Krang Reservoir, Bo Phloi District, Kanchanaburi Province, Thailand (14°32.382′ N, 99°32.670′ E), coll. S. Savatenalinton, 18 October 2018.
- Other localities. See Table 1, coll. S. Savatenalinton.

#### 3.2.5. Measurements (In μm)

Cp (*n* = 3), L = 566–585, H = 370–372, W = 352–365; LV (*n* = 4), L = 533–584, H = 349–394; RV (*n* = 4), L = 520–578, H = 346–382.

#### 3.2.6. Description of Female

- Cp in lateral view (Figure 1A) subtriangular, anterior and posterior ends widely, subequally rounded, dorsal margin strongly arched, maximum height situated about mid-length, ventral margin straight, LV slightly larger than RV, LV overlapping RV anteriorly, posteriorly and ventrally.
- Valve surface (Figure 1B) with smooth spines dispersed and grounded with pits and two types of setae: type 1 (long, thin and smooth seta) and type 2 (long, thin seta with thick base), pore of the latter with two small lateral tubercles.
- Cp in dorsal views (Figure 1C) subovate with evenly curved lateral margins, maximum width situated slightly behind mid-length, posterior end rounded, anterior end more pointed.
- Cp in ventral views (Figure 1D,E): ventral margin of LV with stronger outer list at mid-length, LV margin protruded at mid-length and with a row of setal pores along valve border.
- LV in internal view (Figure 2C,E–G) with anterior and posterior valve margins broadly, subequally rounded, dorsal margin strongly arched, ventral margin somewhat straight. Anterior calcified inner lamella with two inner lists, posterior part with double inner list, appearing well developed, but not oblique. Sockets of tubercles located along valve margin.
- RV in internal view (Figure 2D,H,J) with anterior valve margin widely rounded, posterior valve margin slightly protruded at mid-height, dorsal margin strongly arched, ventral margin somewhat straight. Anterior and posterior selvage slightly inwardly displaced, with tubercles along the outer margin of the selvage anteriorly, ventrally, and posteriorly; marginal tubercles more distinctive on antero-ventral and postero-ventral parts. Anterior calcified inner lamella with one inner list; posterior calcified inner lamella with one inner list.
- A1 (Figure 3A): Seven-segmented, first segment with short dorso-subapical (not reaching tip of next segment) and two long ventro-apical setae (length of short one ca. two-thirds of long one), Wouters organ very small. Second segment ca. two times wider than long, with short dorso-apical seta (reaching one-fourth of next segment) and small Rome organ. Third segment bearing one long dorso-apical (reaching tip of sixth segment) and short ventro-apical setae (not reaching tip of next segment). Fourth segment with two long dorsal setae and two short ventral setae (both reaching tip of next segment). Fifth segment dorsally with two long setae, ventrally with two (one long, one short) setae, the short one reaching the tip of the next segment. Sixth segment with four long apical setae and one short dorsal seta. Seventh segment with three (two long, one shorter) apical setae and a considerably long aesthetasc ya, its length longer than the length of the last four segments; shorter seta reaching mid-length of aesthetasc ya.
- A2 (Figure 3B–D): Basal segment with three subequal, proximal setae and long ventro-apical seta. Exopodite with three (one long, two short) setae, long one reaching mid-length of terminal segment. First endopodite segment with five very long natatory setae (reaching far beyond tips of terminal claws) and shorter accompanying seta (reaching beyond mid-length of penultimate segment), natatory setae with pseudosegmentation; aesthetasc Y long, three-segmented, ventro-apical seta long, extending beyond tip of terminal segment. Penultimate segment short, undivided, distally with 3 large, serrated claws (G1–G3), G2 strongly serrated, apically with concave apex, serration appearing about half length with large teeth apically and gradually reduced in size proximally, aesthetasc y2 short (not reaching tip of terminal segment), z1–z3 setae long, z1 clearly larger than other z setae, z3 longest (reaching tip of claws G1); median part of segment with two (one long, one short) dorsal setae (long one reaching mid-length of G2 claw, length of short one ca. one-third of the long one), and four ventral setae of unequal length (t1–t4), t2 large, claw-like and longest. Terminal segment subquadrate, with two serrated claws (GM, Gm), length of Gm ca. 3/5 of that of GM; with aesthetasc y3, length of aesthetasc y3 ca. two-thirds of that of accompanying seta, g seta not seen.
- Md coxa (Figure 3E): Elongated, distally with rows of teeth and small setae (ventral seta longest), and with one short plumose dorso-subapical seta (reaching row of teeth).
- Md palp (Figure 4A): First segment with two large setae, one long and slender seta and α seta; the latter slender, smooth, with a flagellum-like tip. Second segment dorsally with three unequal long apical setae, shortest reaching the tip of next segment; ventrally with group of three long hirsute setae, one shorter hirsute seta (reaching beyond tip of next segment) and plumose, cone-shaped β seta with remarkably long, flagellum-like ending. Third segment dorsally with group of four unequally long, subapical setae; laterally with apical γ seta and three apical setae, the former plumose (length ca. 2.5 times that of terminal segment); ventrally with two (one long, one short) subapical setae, short one reaching mid-length of terminal segment. Terminal segment subquadrate, bearing three claws and two setae, largest claw appearing strongly robust with hairy ending.
- Mx1 (Figure 4B,C) with two-segmented palp, basal segment of palp dorsally with group of four long, unequal apical setae and long subapical seta; laterally with long subapical seta (not reaching tip of terminal segment), terminal segment elongated (length ca. two times of width), apically with three claws and two setae. Basal seta on third endite long (almost reaching tip of endite). Large bristles on endites claw-like, with spatula-shaped apex and hirsute ending, with the number of large bristles on first to third endites being 2, 2, 2, respectively.
- T1 (Figure 4D): Protopodite with two subequal a-setae, b, c and d setae absent, distally with 10 hirsute apical setae of unequal length, subapical setae absent. Endopodite weakly built palp with one long seta and two unequally shorter apical setae (Figure 4E).
- T2 (Figure 5A) with short, thin d2 seta, d1 absent. Second segment with long e seta (reaching mid-length of fourth segment). Third segment with long f seta (reaching far beyond tip of terminal segment). Fourth segment with short apical g seta. Terminal segment dorsally with apical h1 seta (length ca. one sixth of h2 claw), medially with long, curved, serrated h2 claw with slightly bifurcated tip and ventrally with tiny spine-like h3 seta.
- T3 (Figure 5B) first segment with long d1, d2 and dp setae. Second segment with long apical e seta (reaching mid-length of next segment). Third segment with short f seta (not reaching tip of segment). Terminal segment with short h1 seta, claw-like h2 seta and long reflexed subapical h3 seta, length of the latter ca. two times that of h2.
- CR (Figure 5C) reduced, flagellum-like with triangular base, laterally with short subapical seta (reaching tip of ramus).
- Male unknown.

#### 3.2.7. Remarks

Superficially, *T. baimaii* n. sp. and *T. longispinosa* are similar due to the external morphology of the valves, especially the presence of pits and spines. *Thaicypridopsis baimaii* sp. nov. is primarily distinguished from *T. longispinosa* by short and smooth spines on the valve surface (long and serrated in *T. longispinosa*). The RV posterior margin is protruded in *T. baimaii* sp. nov., while it is rounded in *T. longispinosa*. In addition, the chaetotaxy of the limbs is also different; some significant diagnostic characters are highlighted here. In *T. baimaii* sp. nov., α seta on the Md palp is longer and β seta represents a markedly long flagellum-like tip (absent in *T. longispinosa*). Both species possess large bristles on the Mx1 endites, but the numbers on the first and the second endites are two and two in the new species, whereas they are one and one, respectively, in *T. longispinosa*. The d2 and h3 setae on the T2 are shorter in *T. baimaii* sp. nov. The detailed differences between these two species are provided in Table 2.

#### 3.2.8. Distribution and Ecology

**Figure 6:**
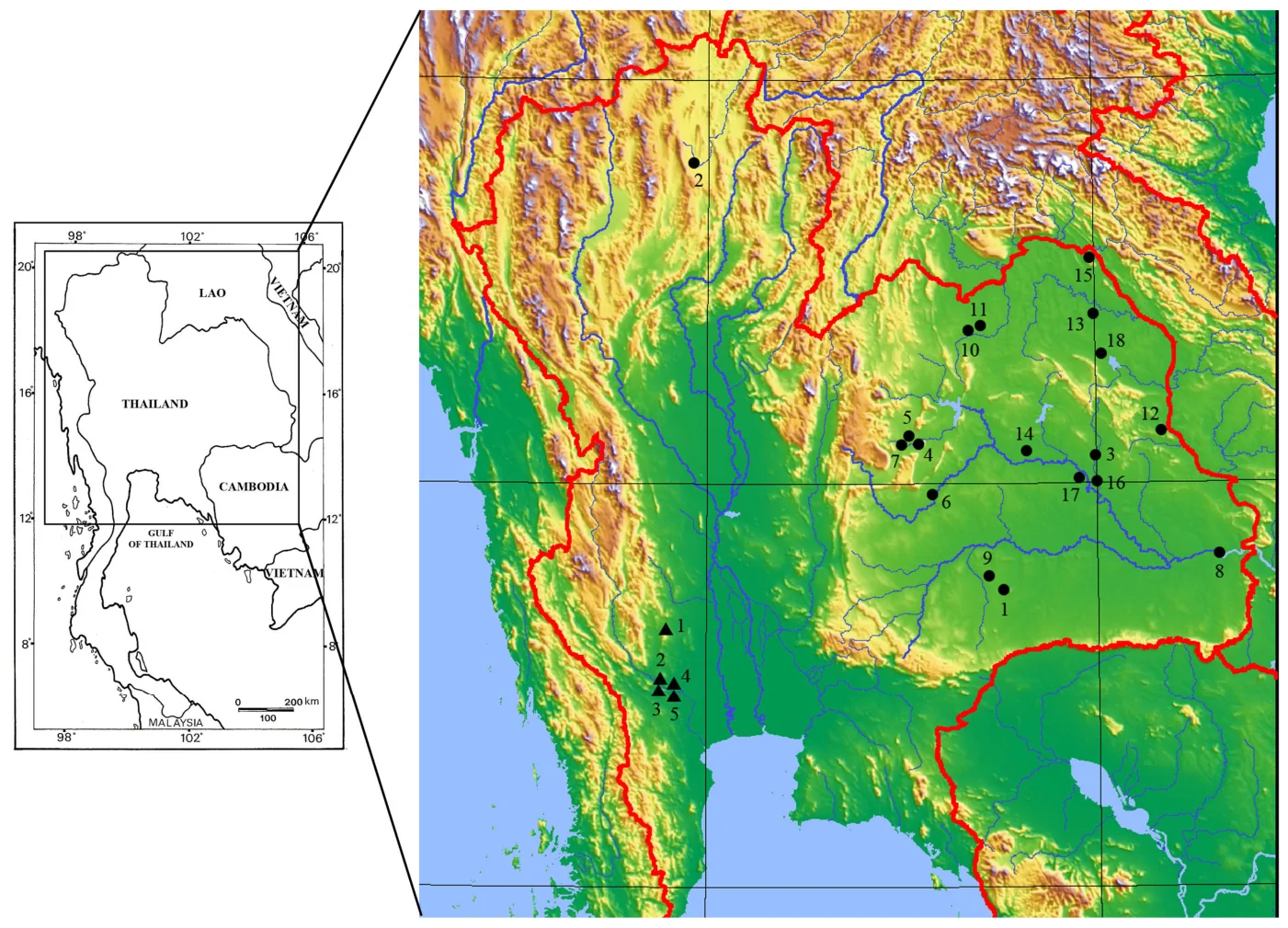
Distribution map of *Thaicypridopsis baimaii* sp. nov. (black triangle) and *Thaicypridopsis longispinosa* Savatenalinton, 2018 (black circle) (modified from [22]). Numbers are shown in Table 1.

**Figure 7:**
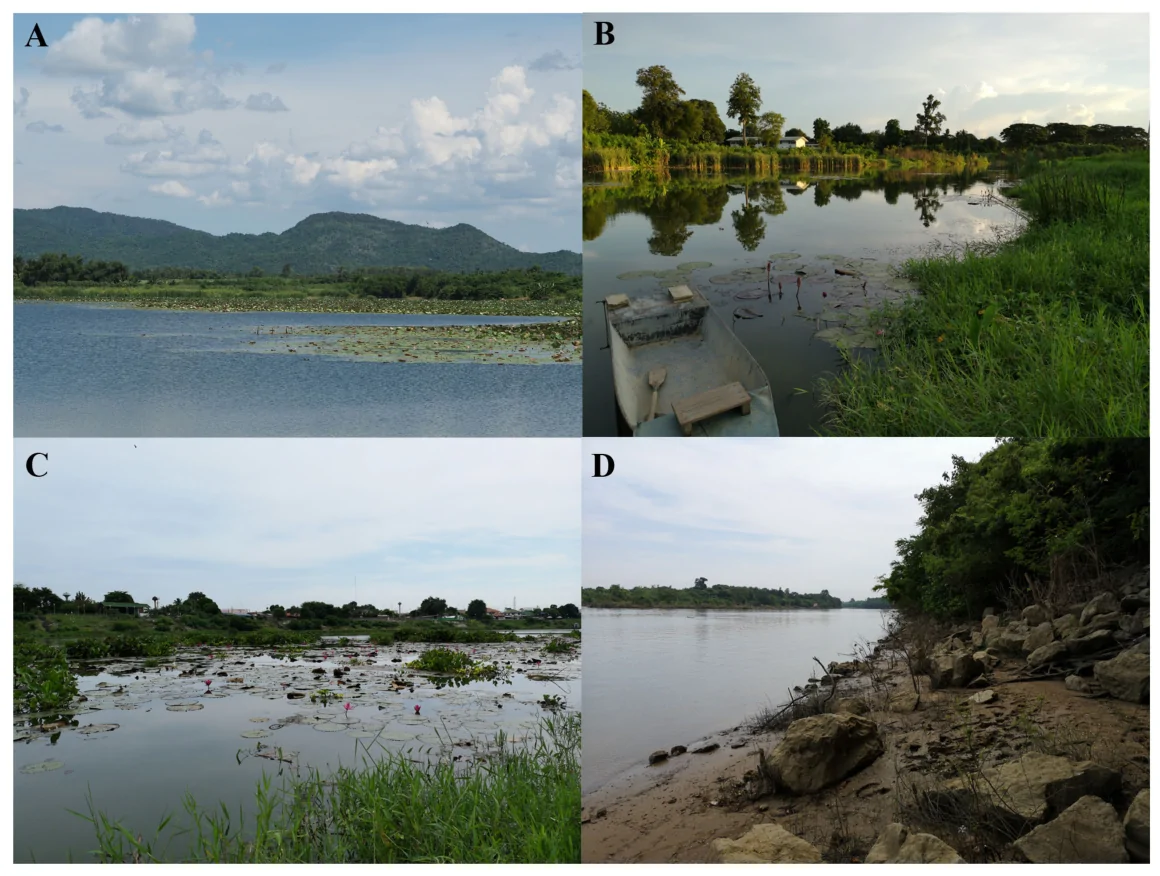
Localities of *Thaicypridopsis baimaii* sp. nov. in Kanchanaburi Province. (**A**) Nong Krang Reservoir, Bo Phloi District. (**B**) A swamp, Muang District. (**C**) A swamp, Tha Muang District. (**D**) Mae Klong River, Muang District.

*Thaicypridopsis baimaii* sp. nov. has so far been found only in five water bodies (natural reservoirs, swamps, and rivers) in Kanchanaburi province in the western part (Figure 6 and Figure 7).

## 4. Discussion

### 4.1. Morphology of Thaicypridopsis and Related Taxa

*Thaicypridopsis* was erected on the basis of a Thai peculiar species, *T. longispinosa*, which shares characters of Cypridopsinae (family Cyprididae) and Oncocypris in Oncocypridinae (family Notodromadidae) [11]. *Thaicypridopsis baimaii* sp. nov. is the second species of the genus and, thus, its discovery not only allows confirmation of generic characters which usually are limited in a monospecific genus, but also provides the specific features. The marginal tubercles of the RV are an indicative feature of the genus, which was recognized on the RV postero-ventral part, based on the description [11]. However, the detailed examination revealed that both species agree in having tubercles located on the anterior, ventral and posterior margins, and more prominent on the postero-ventral part, while the ventral part bears tiny tubercles, which are easily overlooked. The other confirmed characters of the genus *Thaicypridopsis* are the concave tip of the G2 claw on the A2, the largest claw on the Md palp being strongly robust with a hairy ending and the slightly bifurcated tip of the h2 claw on the T2, as they exist in both species.

Pseudosegmentation on natatory setae of the A2 was initially illustrated in Mixtadopsis [23] and, subsequently, in four species of two other cypridopsine genera (*Pseudocypretta* and *Cyprettadopsis*) [10]. Such a feature was also recognized in the new *Thaicypridopsis* species. In addition, after rechecking the type material, this feature was found in *T. longispinosa*. Hence, it is considered a generic character. Thus far, this pseudosegmentation has been exhibited in five genera of the Cypridopsinae: *Cyprettadopsis*, *Mixtadopsis*, *Pseudocypretta*, *Songkhramodopsis* and *Thaicypridopsis*.

One of the significant diagnostic characters of *Thaicypridopsis* is the existence of large modified bristles on all three endites of the Mx1, which make it considerably different from other Cypridopsinae genera. The aspects of these bristles are similar in both species of the genus, appearing spatula-shaped at the apex and hirsute, brush-like at the end, but the numbers are different. In *T. longispinosa*, the first, second and third endites bear two, one and one large bristles, whereas the numbers are two on each endite in *T. baimaii* n. sp. This indicates that the number of such modified bristles is variable within the genus and thus regarded as a specific character which is useful for identification. It should also be noticed that the maximum number on each endite is still restricted to two, which differs from the situation of *Oncocypris* being four or five [24,25,26,27].

**Figure 8:**
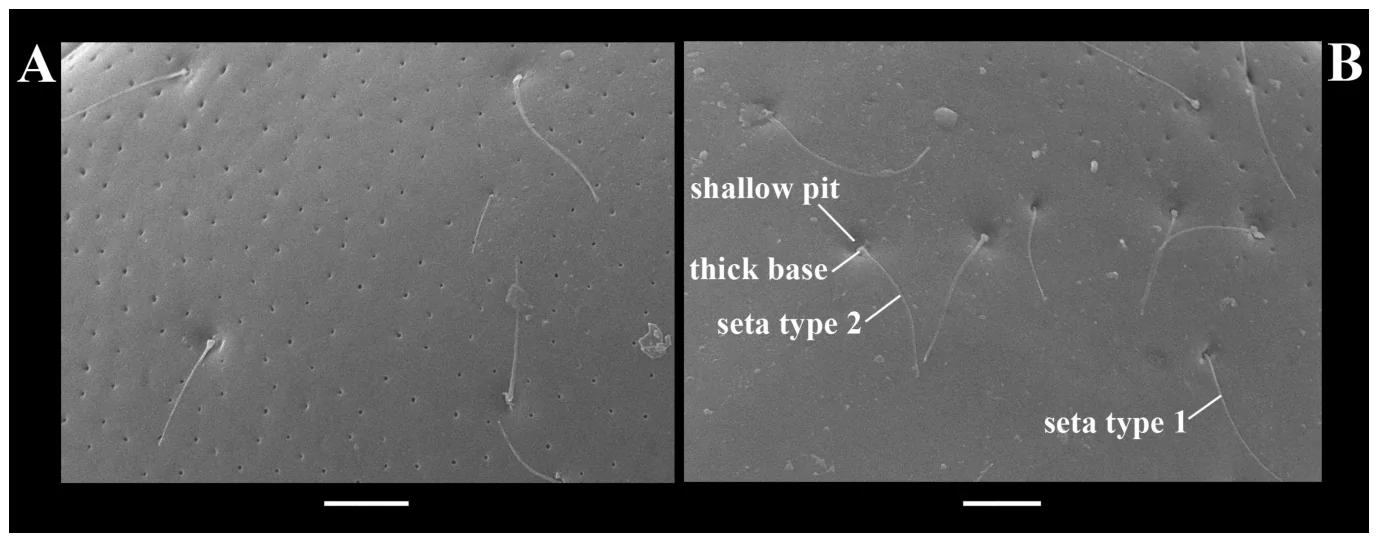
*Songkhramodopsis meischi* Savatenalinton, 2023. (**A**) Valve surface of RV posterior part (MSU-ZOC.355). (**B**) Valve surface of RV anterior part (MSU-ZOC.365). Scale bars: 10 μm (**A**,**B**).

Besides the spines, the valve surface of *T. baimaii* n. sp. is grounded by pits and two types of setae. Like the new species, *T. longispinosa* also displays the same valve sculpture (see Figure 1F). Recently, Savatenalinton & Magnussen [10] demonstrated the valve surface of two Cypridopsinae genera (*Pseudocypretta* and *Cyprettadopsis*) that belong to the tribe Cyprettadopsini. They classified two types of setae as type 1 and type 2. The former was the common form, while the latter was defined by the presence of a thick base and a lateral tubercle at the edge of the pore (see Figure 1F in [10]). Upon examining the type material of *Songkhramodopsis*, a genus in Songkhramodopsini, both seta types were also seen (see Figure 8). The setae on the valve surface of *Thaicypridopsis* are similar to those of Cyprettadopsini and Songkhramodopsini members. Nevertheless, the pore rime of seta type 2 is accompanied by two lateral tubercles in *Thaicypridopsis*, whereas one lateral tubercle is recognized in Cyprettadopsini and is lacking in Songkhramodopsini. It should also be noted that the seta type 2 of Songkhramodopsini is located in pits, which differs from that of the two other tribes (Cyprettadopsini and Zonocypridini) appearing on the ground level. The resemblance of the setae in the three tribes would suggest their close relationship rather than that of other cypridopsine tribes. In addition, it could be regarded as a convergent aspect that evolved independently in these three lineages.

Due to the fact that the two *Thaicypridopsis* species possess geographic differences (see discussion below), the different morphologies are possibly considered as an ecotype of a species. The variability of external valve features can be caused by the environment, as seen in some ostracod species (e.g., *Limnocythere inopinata* (Baird, 1843) and *Cyprideis torosa* (Jones, 1985) (see [28,29]), but soft-part morphologies, which are more conservative characters, can confirm them as separate species.

### 4.2. Distribution

*Thaicypridopsis* is an endemic and rare genus of Thailand. According to a total of 424 sampled localities in four parts of the country, *T. longispinosa* was recorded from 18 localities (Figure 6). Seventeen of them were situated in several provinces in the northeast, while one was in the north ([11], present study). Unlike *T. longispinosa*, *T. baimaii* sp. nov. is more restricted; it has only been found in five water bodies in one province, Kanchanaburi, in the west. It is most likely that the dispersal of the new species is related to the Kwae River, as all five localities lie in the Khwae River Basin. However, in the west, 45 localities in five provinces were inspected, which is a small number compared to 322 sampled sites in 19 provinces of the northeast. Further investigation in the west, or even in other poorly studied parts of the country, would clarify the actual distribution of *T. baimaii* sp. nov. and also *T. longispinosa*. Nonetheless, to date, *T. baimaii* sp. nov. has never been found in the extensive surveys of the northeast, which plausibly suggests that it does not inhabit this part and originates from other areas. This phenomenon is possibly caused by the geographic barrier due to the fact that the northeast is isolated from the central and western parts by the Phetchabun and Dong Phaya Yen Mountain Ranges and also the large Chao Phraya River Basin. The Phetchabun Mountain Range forms a natural boundary between the Chao Phraya River central plain and the Khorat Plateau of the northeast [30,31]. The speciation of this lineage may have occurred via allopatric mode, approximately after the late Permian or Triassic periods (280–240 mya) when the Phetchabun Mountains were elevated by continental drift (e.g., [32,33,34]).

### 4.3. Ecology

Several species of ostracods are common in Thailand (e.g., *Strandesia kraepelini* (G.W. Müller, 1906), *Pseudocypretta maculata* Klie, 193), recorded from many kinds of waters, but some others are specific to habitats. For example, *Bradleytriebella tuberculata* (Hartmann, 1964) and *Notodromas sinensis* Neale & Zhao, 1991, have so far been found only in rice fields, the human-made and temporary waters [10,35]. However, in other countries, these two species also occurred in other kinds of habitats (see, e.g., [36,37,38,39]). In the case of *Thaicypridopsis*, it is noteworthy that the habitat types of both species are similar; they inhabited reservoirs, swamps and rivers (see Figure 7, Table 1). All are natural and permanent water bodies. To date, no human-made or temporary waters (e.g., ponds, irrigation canals, ditches, rice fields) have harbored the *Thaicypridopsis* species and this could imply their poor adaptation, which possibly resulted in their colonization and distribution. Nonetheless, as the survey may have under-sampled rice fields, canals in the west, and temporary habitats may dry prior to sampling, further investigation is needed to confirm this hypothesis. *Thaicypridopsis longispinosa* was known from freshwater with the pH, Temp and Cond in the range of 6.70–9.03, 21.8–30.7 °C and 96–176 μScm^−1^, respectively, while *T. baimaii* sp. nov. inhabited in the ranges of pH 7.53–8.27, Temp 27.3–30.5 °C and Cond 109–456 μScm^−1^.

## 5. Conclusions

*Thaicypridopsis*, a rare and endemic genus of Thailand, is no longer monospecific due to the discovery of *T. baimaii* sp. nov. in the present study. The new species differs from the type species, *T. longispinosa*, in the morphology of both soft parts and valves. Some specific/generic characters are introduced herein. The examination of valve sculpture represents the affinity of three tribes of the Cypridopsinae (Cyprettadopsini, Songkhramodopsini and Zonocypridini). Based on extensive surveys in the northeast, *T. baimaii* sp. nov. is plausibly unpopulated in this part and originates in other areas. At present, the two *Thaicypridopsis* exhibit different geographic distributions, which would be related to vicariant isolation. As poorly studied areas remain in, e.g., the west and the central part of the country, a larger spatial scale including diverse types of waterbodies is required to confirm their distribution and habitats.

## Figures and Tables

**Table 1:** List of localities of *Thaicypridopsis baimaii* sp. nov. and *Thaicypridopsis longispinosa* Savatenalinton, 2018, * type locality.

Number	Locality	Coordinates	Sampling Date	Ind. No.	pH	Temp (°C)	Cond (μScm^−1^)
*Thaicypridopsis baimaii* sp. nov.							
1 *	Nong Krang Reservoir, Bo Phloi District, Kanchanaburi Province	14°32.382′ N, 99°32.670′ E	18 October 2018	122	7.53	28.7	375
			12 March 2019	44	7.81	27.3	188
2	Khwae Yai River, Muang District, Kanchanaburi Province	14°01.609′ N, 99°31.425′ E	12 March 2019	1	7.96	28.5	172
3	A swamp, Muang District, Kanchanaburi Province	13°58.216′ N, 99°30.574′ E	18 October 2018	183	7.99	30.5	344
4	A swamp, Tha Muang District, Kanchanaburi Province	13°58.215′ N, 99°37.862′ E	19 October 2018	24	8.27	27.6	456
			13 March 2019	194	7.97	28.0	109
5	Mae Klong River, Muang District, Kanchanaburi Province	13°57.121′ N, 99°37.725′ E	19 October 2018	5	7.94	27.5	281
*Thaicypridopsis longispinosa* Savatenalinton, 2018							
1 *	Huai Jorakae Mak (reservoir), Muang district, Buriram Province	14°54.683′ N, 103°03.117′ E	4 October 2010	17	8.27	27.6	117
2	Kwan Phayao (lake), Muang District, Phayao Province	19°09.550′ N, 99°54.600′ E	6 October 2007	2	7.10	26.8	-
3	Lam Phayom (reservoir), Roi-Et Province	16°17.283′ N, 103°59.767′ E	20 February 2010	1	6.70	25.5	-
4	Nong PagPung (swamp), Phu Kiew District, Chaiyaphum Province	16°22.450′ N, 102°08.033′ E	30 October 2010	7	7.32	26.1	163
5	Nong Sammuen (swamp), Phu Kiew District, Chaiyaphum Province	16°24.450′ N, 102°02.030′ E	30 October 2010	1	7.22	26.1	176
6	Lam Nam Kam (rivulet), Muang, Chaiyaphum Province	15°46.617′ N, 102°13.300′ E	3 October 2010	3	9.03	30.0	164
7	Huai Pao (river), Kaset Sombun District, Chaiyaphum Province	16°23.050′ N, 101°58.780′ E	9 October 2007	1	7.20	29.6	-
8	Kang Sapue (river), Phiboon Mangsahan District, Ubon Ratchathani Province	15°14.617′ N, 105°14.617′ E	24 October 2010	1	7.18	29.0	123
9	Chon Pratan Reservoir, Lampaimas District, Buriram Province	15°01.083′ N, 102°50.483′ E	8 February 2011	2	7.43	25.8	99
10	Nong Kae (swamp), Muang district, Udon Thani Province	17°27.983′ N, 102°48.650′ E	31 January 2011	2	7.60	30.6	-
11	Huai Luang (reservoir), Muang district, Udon Thani Province	17°28.333′ N, 102°49.200′ E	31 January 2011	2	7.40	28.7	-
12	Nong Lom (swamp), Don Tan District, Mukdahan Province	16°24.300′ N, 104°51.500′ E	19 December 2011	11	7.54	21.8	-
13	Nam Chan Reservoir, Akat Amnuai District, Sakon Nakhon Province	17°35.970′ N, 104°0.905′ E	18 November 2021	2	6.94	30.7	-
14	Nong Bua (swamp), Kantharawichai District, Maha Sarakham Province	16°19.250′ N, 103°17.784′ E	22 October 2021	3	-	-	-
			22 January 2022	32	-	-	-
15	Huai Bang Bat Reservoir, Bueng Kan Province	18°16.614′ N, 103°52.872′ E	19 March 2024	2	-	-	-
16	Huai Pla Khun Reservoir, Selaphum District, Roi Et Province	15°57.934′ N, 104°0.471′ E	23 October 2024	9	7.63	28.7	96
17	Kut Khaen (swamp), Thawat Buri District, Roi Et Province	16°0.144′ N, 103°48.083′ E	23 October 2024	1	7.69	29.7	137
18	Nong Han Lake, Muang District, Sakon Nakhon Province	17°15.059′ N, 104°10.689′ E	16 March 2023	1	-	-	-

**Table 2:** Comparison between *Thaicypridopsis baimaii* sp. nov. and *Thaicypridopsis longispinosa* Savatenalinton, 2018.

Characters	*Thaicypridopsis longispinosa*	*Thaicypridopsis baimaii* sp. nov.
Spine on valve surface	long, serrated	short, smooth
Posterior tip of RV	rounded	protruded
A1 Wouters organ	absent	present
A1 third segment: dorsal apical segment	long (reaching tip of sixth segment)	longer (reaching beyond tip of sixth segment)
A1 terminal segment: shortest seta	less than half-length of ya	half-length of ya
A2 penultimate segment: two dorsal setae	length of short seta ca. 2/3 of that of long seta	length of short seta ca. 1/4 of that of long seta
A2: y3	length ca. half of accompanying seta	length ca. 2/3 of accompanying seta
A2: z2	long (reaching far beyond tip of claw G2)	short (reaching 2/3 of claw G2)
A2 exopodite: longest seta	long (reaching tip of penultimate segment)	shorter (not reaching tip of penultimate segment)
Md palp: α-seta	pointed tip	long flagellum-like tip
Md palp: β-seta	pointed tip	considerably long flagellum-like tip
Mx1 first to third endite: number of large bristles	1, 1, 2	2, 2, 2
Mx1 palp: lateral seta on basal segment	long (slightly beyond tip of terminal segment)	shorter (not reaching tip of terminal segment)
T2: d2 seta	long (reaching beyond tip of segment)	short (not reaching tip of segment)
T2: h1 seta	length ca. 1/4 of that of h2 claw	length ca. 1/5 of that of h2 claw
T2: h3 seta	long (reaching far beyond tip of segment)	remarkably short (reaching tip of segment)
T3: dp seta	long (reaching tip of next segment)	shorter (reaching 2/3 of next segment)
CR: lateral seta insertion	close to tip of ramus	far from tip of ramus
Distribution	wide distribution in the northeastern part	restricted distribution in Kanchanaburi Province, western part
Habitat type	natural reservoir, lake, swamp, river	natural reservoir, swamp, river

## Data Availability

The original contributions presented in this study are included in the article. Further inquiries can be directed to the author.

## Abbreviations

The following abbreviations are used in this manuscript:

A1	first antenna
A2	second antenna
Cond	conductivity
Cp	carapace
CR	caudal ramus
H	height of valves
Ind. no.	number of individuals
L	length of valves
LV	left valve
Md	mandibula
MSU-ZOC.	Ostracod Collection of Faculty of Science, Mahasarakham University
Mx1	maxillula
mya	million years ago
μm	micrometer
R	Rome organ
RV	right valve
T1	first thoracopod
T2	second thoracopod
T3	third thoracopod
Temp	temperature
W	width of carapace
wo	Wouters organ
